# Deciphering chemotaxis pathways using cross species comparisons

**DOI:** 10.1186/1752-0509-4-3

**Published:** 2010-01-11

**Authors:** Rebecca Hamer, Pao-Yang Chen, Judith P Armitage, Gesine Reinert, Charlotte M Deane

**Affiliations:** 1 Department of Statistics, University of Oxford, Oxford, UK; 2 Oxford Centre for Integrative Systems Biology University of Oxford, Oxford, UK; 3 Department of Biochemistry, University of Oxford, Oxford, UK; 4 Department of Molecular, Cell and Developmental Biology, University of California, Los Angeles, USA

## Abstract

**Background:**

Chemotaxis is the process by which motile bacteria sense their chemical environment and move towards more favourable conditions. *Escherichia coli *utilises a single sensory pathway, but little is known about signalling pathways in species with more complex systems.

**Results:**

To investigate whether chemotaxis pathways in other bacteria follow the *E. coli *paradigm, we analysed 206 species encoding at least 1 homologue of each of the 5 core chemotaxis proteins (CheA, CheB, CheR, CheW and CheY). 61 species encode more than one of all of these 5 proteins, suggesting they have multiple chemotaxis pathways. Operon information is not available for most bacteria, so we developed a novel statistical approach to cluster *che *genes into putative operons. Using operon-based models, we reconstructed putative chemotaxis pathways for all 206 species. We show that *cheA-cheW *and *cheR-cheB *have strong preferences to occur in the same operon as two-gene blocks, which may reflect a functional requirement for co-transcription. However, other *che *genes, most notably *cheY*, are more dispersed on the genome. Comparison of our operons with shuffled equivalents demonstrates that specific patterns of genomic location may be a determining factor for the observed *in vivo *chemotaxis pathways.

We then examined the chemotaxis pathways of *Rhodobacter sphaeroides*. Here, the PpfA protein is known to be critical for correct partitioning of proteins in the cytoplasmically-localised pathway. We found *ppfA *in *che *operons of many species, suggesting that partitioning of cytoplasmic Che protein clusters is common. We also examined the apparently non-typical chemotaxis components, CheA3, CheA4 and CheY6. We found that though variants of CheA proteins are rare, the CheY6 variant may be a common type of CheY, with a significantly disordered C-terminal region which may be functionally significant.

**Conclusions:**

We find that many bacterial species potentially have multiple chemotaxis pathways, with grouping of *che *genes into operons likely to be a major factor in keeping signalling pathways distinct. Gene order is highly conserved with *cheA-cheW *and *cheR-cheB *blocks, perhaps reflecting functional linkage. CheY behaves differently to other Che proteins, both in its genomic location and its putative protein interactions, which should be considered when modelling chemotaxis pathways.

## Background

Chemotaxis is the process by which motile bacteria move towards more favourable conditions by sensing their chemical environment. It is of significant medical interest, as many pathogenic bacteria depend on chemotaxis and motility to invade their hosts. For example, *Helicobacter pylori*, which colonizes the mucus lining of the stomach, has a chemotactic response to gastric mucin [1]. Biofilm development depends on chemotaxis, and their formation in the lungs of cystic fibrosis patients and on medical implants can have serious consequences [2,3]. Chemotaxis is also essential for symbiotic associations of bacteria, for example the colonization of wheat roots by the nitrogen fixing bacterium *Azospirillum brasilense *[4]. In addition, chemotaxis is the canonical system used to study signalling pathways in systems biology, due to its relative simplicity for modelling purposes, and the ease with which it can be studied experimentally. A detailed, quantitative understanding of chemotaxis pathways would pave the way for the study of other, more complex signalling systems.

**Figure 1:**
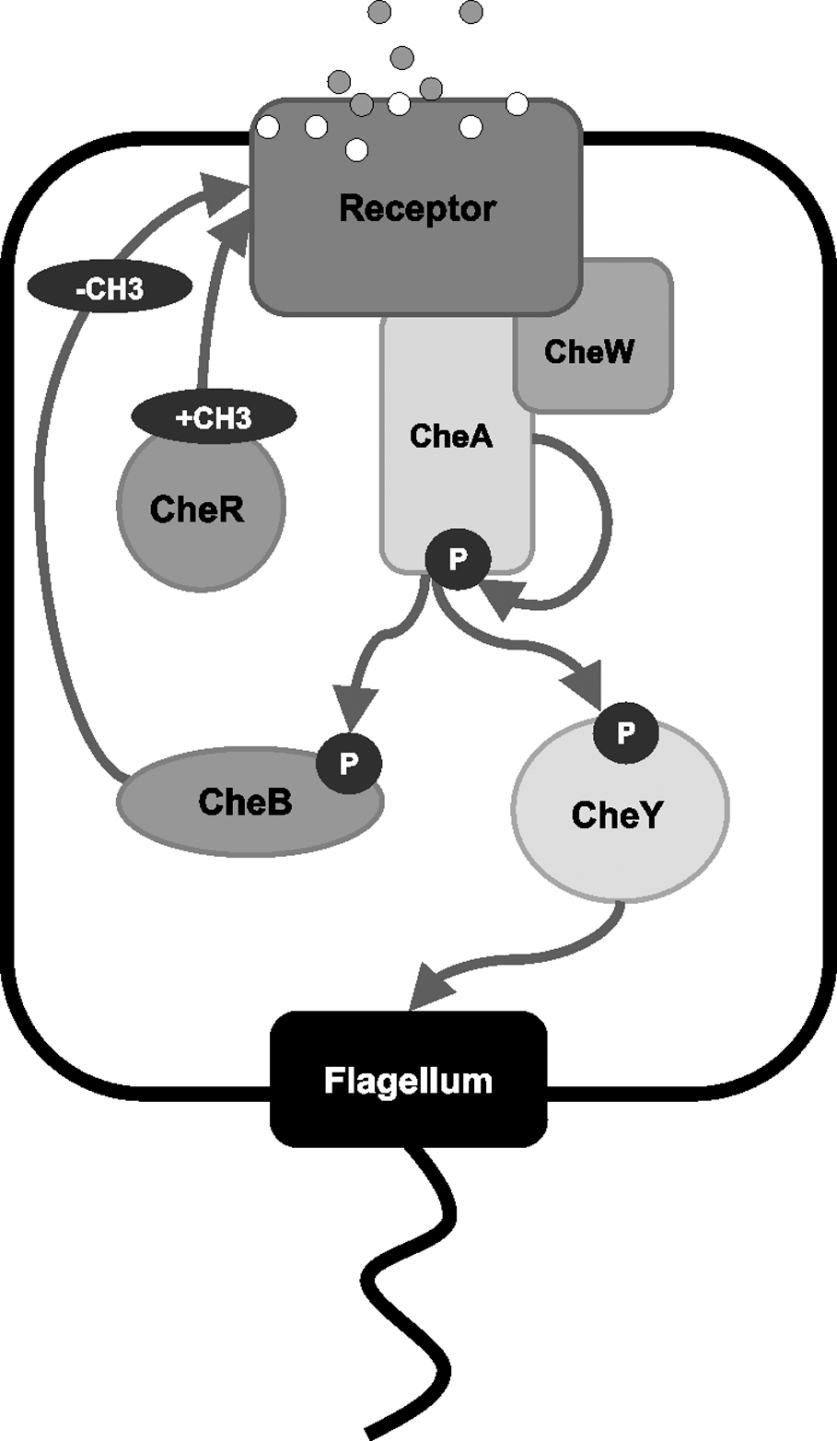
**Schematic representation of the core chemotaxis pathway**. Reduction in chemoattractant or increase in chemorepellant is sensed by membrane-bound methyl-accepting chemotaxis receptors. This activates CheA and triggers transphosphorylation. The phosphoryl group is then transferred to either CheY or CheB. Phosphorylated CheY is able to diffuse and bind to the FliM component of the flagellar motor, resulting in a change in motor rotation from counter-clockwise to clockwise. This causes a switch from smooth swimming to tumbling and hence a change in swimming direction. Phosphorylated CheB competes with the constitutively active methyltransferase CheR, causing demethylation of the receptors, which reduces their ability to activate CheA. This resets the rate of direction changing to pre-stimulus levels, a process known as adaptation.

*Escherichia coli *chemotaxis has been extensively studied and is known to use a single 2-component histidine protein kinase-dependent signal transduction pathway consisting of the Che proteins A, B, R, W, Y and Z [5]. Any reduction in chemoattractant or increase in chemorepellent in the periplasm is sensed by membrane-spanning methyl-accepting chemotaxis receptors (MCPs) which are linked via CheW to a dimeric histidine protein kinase, CheA, to form a large, polar, quaternary protein complex. On activation, the monomers of CheA transautophosphorylate and the phosphoryl group is then transferred to one of two response regulators, either CheY or CheB. Phosphorylated CheY is able to diffuse and bind to the FliM component of the flagellar motor, resulting in a change in motor rotation from counter-clockwise to clockwise. This causes a switch from smooth swimming to tumbling, allowing the bacteria to change direction. The motor has a baseline stochastic switching frequency in the absence of any stimulation, but binding of phosphorylated CheY increases this rate. CheY is dephosphorylated by CheZ, which terminates the signal. The constitutively active methyltransferase CheR transfers methyl groups to the MCPs, increasing their ability to activate CheA. Phosphorylated CheB competes with CheR, by removing methyl groups from the MCPs, so reducing their ability to activate CheA. This decreases the rate of CheA transphosphorylation and so resets the rate of direction changing to pre-stimulus levels, resulting in adaptation. A schematic of a generalised chemotaxis pathway, consisting of the core chemotaxis proteins (CheA, CheB, CheR, CheW and CheY) is shown in Figure 1. CheZ is not included here as many species do not have a CheZ homologue.

*E. coli *chemotaxis is a relatively simple biological system which is conserved across many bacterial and archaeal species, and has inspired the development of numerous mathematical models of chemotaxis [6,7]. However, some bacteria have multiple homologues of the Che proteins which probably form more than one chemotaxis pathway. *Rhodobacter sphaeroides *is a well studied example of a species with multiple chemotaxis pathways. It has several homologues of *E. coli *CheA, B, R, W and Y proteins but none of CheZ. There are three operons encoding the majority of the chemotaxis genes (CheOp1, CheOp2 and CheOp3), as well as other unlinked loci encoding chemoreceptors and CheY homologues [8]. CheOp2 and CheOp3 are essential for chemotaxis in the laboratory, while the physiological role of CheOp1 has not yet been established [9-13]. Of the 13 chemoreceptor homologues, 4 lack transmembrane regions and are referred to as transducer-like proteins (Tlps). These are cytoplasmic and sense the metabolic state of the cell rather than the exterior environment. Studies have shown the presence of two discrete protein clusters in the cell, with the proteins encoded by CheOp2 localising with the MCPs at the cell pole, and proteins encoded by CheOp3 localising with cytoplasmic Tlps [14]. Chemotaxis requires signals from both these clusters to be integrated to produce a response at the single flagellar motor. Exactly how these clusters are formed and targeted to the correct position in the cell is still the subject of active research [15], but it is known that the PpfA protein, which is homologous to bacterial type I DNA partitioning factors and is encoded in CheOp3, is critical for correct partitioning of the cytoplasmic protein clusters on cell division [16].

CheA has 5 domains designated P1-P5 [17,18]. P1 is the histidine-containing phosphotransfer (HPt) domain, P2 contains the binding site for CheY and CheB, P3 is the dimerization domain, P4 is the kinase domain which phosphorylates a conserved histidine in P1, and P5 binds to CheW and the receptors. *R. sphaeroides *contains two classical CheA homologues, and two with missing domains. CheA3 has only P1 and P5 domains, separated by a 794 residue long linker region which includes a CheY6-specific phosphatase domain [19], and CheA4 has only domains P3, P4 and P5 [9]. The reason for the presence of such unusual homologues and their prevalence among other bacterial species is currently unknown.

Activated CheA transfers the phosphoryl group from its P1 domain to the response regulator, CheY. CheYs belong to a group of proteins termed single domain response regulators (SDRRs), but differ from classical SDRRs as they do not include an output domain. Genomes can encode a large number of SDRRs and their annotation as CheY-encoding genes usually depends on their genomic context. *R. sphaeroides *has at least 6 CheY homologues and a minimum of two are necessary for chemotaxis; CheY6 and either of CheY3 or CheY4. However, although all of CheY1-6 can bind to FliM, only CheY6 is capable of stopping the flagellar motor, the mechanism by which swimming direction is changed in this species [20]. CheY6 is also unusual in that it autodephosphorylates ten times faster than CheY1-5 and *E. coli *CheY [21]. It is also possible that CheY1-5 act as phosphate sinks to aid signal termination [22,23], and/or they may compete with CheY6 for binding to FliM [8]. Although CheY6 is the major motor-binding CheY, and is annotated as CheY because of its genome context, it is in fact more similar to the REC domain of *E. coli *CheB than to *E. coli *CheY.

While chemotaxis requires Che proteins, not all Che protein homologues are specialised for chemotaxis. Some species, such as *Pseudomonas aeruginosa *and *Myxococcus Xanthus*, contain operons encoding Che protein homologues which have become specialised for an alternative function such as twitching motility or controlling gene expression during development [24-26].

This work uses operon information to predict chemotaxis pathways from the genetic sequence of a species. Unfortunately, experimentally-based information about operons is not readily available for a large number of bacterial genomes, so here we use a novel statistical procedure based on the proximity of genes on the genome to predict clusters of *che *genes. These clusters are referred to from here on as operons, but it should be recognised that these are computational predictions. Previous approaches use cut-offs on operon lengths [27], or the distance between two adjacent genes [28,29]*e.g*. the Gene Gap Method [30]. However, methods based on operon lengths could potentially result in the distance of adjacent genes in an operon being larger than the distance between two operons. On the other hand, methods based on the distance between two adjacent genes may result in large operons in species with short genomes. In both approaches, the selection of cut-offs is essential, as one fixed cut-off may not apply to all genomes, or even to all operons, due to the large variety of genome sizes across species. Our method overcomes these problems using a multivariate normal clustering method based on the Akaike Information Criterion (AIC) [31].

In building our models of chemotaxis pathways, we assume that CheB, CheR, CheW and CheY are all functionally linked to CheA. We refer to all these functional linkages as 'Putative Interactions' (PIs) for simplicity. Physiologically, CheA directly interacts with CheW, CheB and CheY and has an indirect interaction with CheR, via the chemotaxis receptors. Two putatively interacting Che proteins may be encoded by genes either in the same operon, which we call a within-operon PI, or by genes in two different operons, which we call an across-operon PI. Note that the term 'interaction' does not imply a gene-gene interaction, but rather is shorthand for the fact that the encoded proteins are functionally linked and putatively interact. To date, there is still very little information about cross-talk between chemotaxis proteins encoded by different operons, so we consider five assumptions to construct parsimonious models for chemotaxis pathways from our predicted operons: (i) All Che proteins are part of a complete set required to operate chemotaxis *i.e*. all Che proteins are part of a functioning chemotaxis pathway. This is an idealised assumption, as some Che proteins may serve alternative functions. (ii) The proteins belonging to one pathway 'attempt' to be distinct from those belonging to other pathways. This is supported by the known pathways observed in species such as *P. aeruginosa *[32] and also allows simpler controls within a pathway. (iii) PIs tend to happen between proteins encoded within the same operon in preference to between proteins encoded in different operons, as genes within the same operon are in close proximity and are co-transcribed from the same promoter. (iv) The ranking of probabilities of within-operon PIs and of across-operon PIs is identical in every species, since Che proteins maintain the same functions across species. This may reflect horizontal gene transfer of whole pathways between species. Note that the probabilities themselves do not have to be identical, only the ranking. (v) A chemotaxis pathway tends to minimise the number of operons its proteins are encoded in. This is based on the biological conjecture that the fewer operons used, the simpler the control mechanism. Using our models, chemotaxis pathways are predicted for each of the 206 species.

We demonstrate that the organisation of genes into operons is not arbitrary, and observe that *cheA *and *cheW *are frequently adjacent within operons, as are *cheB *and *cheR*. This may reflect the close relationship of the encoded proteins, for example in forming a protein complex or in the adaption mechanism, respectively, and possibly the need for a strict stoichiometric relationship [33]. We find that the distribution of *cheY *in operons is different from the other *che *genes and that CheY PI behaviour is different to that of other Che proteins. This finding is in line with those of Wuichet *et al*. [34], where the problem of distinguishing CheY proteins in a set of stand-alone REC domains is discussed. As CheY proteins have been shown to regulate both flagellar and pili-based motility, Wuichet *et al*. also argue for a special treatment of CheY. With the caveat that identifying a CheY homologue as being involved in a chemotaxis pathway is problematic, models of chemotaxis which treat CheY differently to the other Che proteins may therefore be most appropriate. We also observe that the presence of *ppfA *homologues within *che *operons is widespread, suggesting that cytoplasmic clusters of Che proteins may be common to many bacterial species. In species with multiple Che homologues, grouping of *che *genes into operons and localization of Che proteins into clusters in the cell are likely to be the major factors determining separation of chemotaxis pathways. Finally, our predictions of chemotaxis pathways not only closely match the available interactions reported in the literature [5,8,32,35] but they also suggest pathways for hitherto less studied species.

## Methods

### Data

Protein sequences in Fasta format from all 833 complete bacterial and archaeal genomes available at the NCBI [36] in February 2009 were downloaded and a non-redundant set of 523 genomes was created by removing multiple strains of the same species.

*E. coli *Che proteins were used as query sequences to search each proteome using BLAST version 2.2.18 [37,38]. Default settings were used but the low complexity filter was turned off and the top 20 000 hits were output, ensuring all hits were retrieved. Results in XML format were filtered using custom PERL scripts to maximise the probability that hits were true Che homologues (Table 1). Filter criteria were refined after manual inspection of initial results to ensure true hits were not being rejected. For example, some CheW hits were originally rejected as they were much longer than *E. coli *CheW. However, on inspection many of these were annotated as CheW homologues and CDART [39] showed no other domains, so the maximum hit length accepted was increased. Distinguishing CheY proteins from non-CheY single domain response regulators (SDRRs) is impossible from sequence alone, but using the strict cut-offs shown in Table 1 reduces this problem.

**Table 1:** BLAST filtering criteria

Protein	Query sequence length	Max hit length	**Domains**^**†**^	**Domains in *E. coli *from CDART **[39]	BLAST 'query from' criteria	BLAST 'query to' criteria
CheA^‡^	654	-	All	1-654	<400	>610

			P4	370-505	>340 & <400	>475 & <535

			P5	509-640	>480 & <540	>610

						

CheB	349	450	All	1-349	<30	>310

			1	7-108	<30	>90 & <160

			2	158-340	>130 & <190	>310

						

CheR	286	415	All	1-286	<40	>250

			1	23-78	<50	>50 & <110

			2	92-283	>60 & <210	>250

						

CheW^§^	167	240	All	1-167	-	-

						

CheY	129	176	All	1-129	<30	>100

						

CheV*	303	400	All	1-303	<40	>250

			1	16-153	<40	>120 & <200

			2	178-301	>140 & <210	>250

						

CheZ	214	300	All	1-214	<30	>180

^†^If more than one domain is present in the query sequence, hits with multiple HSPs in the BLAST output were checked to see if each domain was found separately, using the criteria listed.

^‡^CheA homologues had to contain at least domains P4 and P5. Note that this means CheA3 from *R. sphaeroides *is rejected, as it only contains domains P1 and P5.

^§^No query from or query to restrictions were set for CheW, as this led to the rejection of too many true CheW homologues.

*Query sequence was from *Bacillus subtilis*

Of the 523 genomes examined, only 117 (22%) encode a CheV homologue and 102 (20%) encode a CheZ homologue. In contrast, at least 46% of species have a homologue of CheA, B, R, W and Y (Table 2). We therefore eliminated CheV and CheZ from our analyses, and consider CheA, B, R, W and Y to be the five 'core' Che proteins. In this study, a number of other ancilliary proteins identified as important for chemotaxis in some species have been ignored (*e.g*. CheC, CheD and CheX) as these are found in an even more limited set of species. We have concentrated on the genes found across multiple species that are likely to be core to chemotaxis. The same core proteins are chosen by Kirby [24], while Wuichet *et al*. [34] only consider CheA, CheW and CheY as essential, arguing that occasionally CheB and CheR may be absent [40,41].

**Table 2:** Distribution of *che *genes across all species studied

*che *gene	Number of *che *genes	Number of species	Isolated *che *genes*	Species with isolated *che *genes
CheA	335 (367)	206 (238)	2	2

CheB	352 (408)	206 (249)	19	17

CheR	340 (395)	206 (251)	44	42

CheW	525 (625)	206 (274)	126	83

CheY	1252 (1822)	206 (358)	693	169

CheV	175 (209)	92 (117)	110	68

CheZ	96 (105)	94 (102)	7	7

Numbers in brackets refer to homologues found in all 523 bacterial genomes examined. All other numbers are taken from those species with at least one of each of the 5 core Che proteins.

*Isolated *che *genes refer to *che *genes that are not in close proximity to other *che *genes.

Genomic locations of Che protein homologues in each species were found by searching the bacterial ptt files [42] for corresponding gi accession numbers. Of the 485 species which had a BLAST hit to at least one of CheA, B, R, W, Y, V or Z, *che *genes in 474 species could be located in ptt files (Additional file 1).

206 of these 474 genomes were found to contain at least one homologue of each of the core *E. coli *chemotaxis genes (encoding CheA, B, R, W and Y). The *che *genes from these genomes were grouped into operons using the statistical approach described next. All statistics are based on this data set.

### Assigning genes to operons

For most organisms, operon information is not available. Hence a standard statistical clustering approach for assigning genes to operons is employed, see [43]. Here, there is biological information to be taken into account. The candidate operons are generated from genes which are on the same DNA strand with no intervening *che *genes transcribed in the opposite direction [27]. Intuitively, the maximal distance between two adjacent genes in the same operon should be smaller than the minimal distance between any two adjacent operons; this is made a requirement for the clustering algorithm. Noting that the largest gap between *che *genes in the *E. coli *chemotaxis operon is ~3,500 base pairs, the clustering algorithm assumes that when two *che *genes are within 3,500 base pairs, then these two genes are in the same operon. As the shortest distance between two genes from different *che *operons in *R. sphaeroides *is ~100,000 base pairs, adjacent genes are separated into two operons if their distance is greater than 100,000 base pairs. The resulting assignment of genes to operons is robust with respect to changes of this parameter (100,000 base pairs) within a reasonable range (data not shown).

The coarse assumptions are that genes within an operon have a normally distributed location across the genome, with operon-specific means. The estimation of the number of operons and their gene contents then follows analogously from estimating the number of components in a mixture of normal variables, and assigning the observations to different components of this mixture.

A statistical heuristic assumes that the position of genes, given by their centre points, are approximately independent and normally distributed with means depending on the (unknown) operon they belong to, but with the same, unknown, variance [43]. This is an approximation based on the fact that genes are short in comparison with the length of the genome.

Suppose that an assignment of *n *genes into *k *operons has been constructed; let *n*_1 _be the number of genes assigned to the *l*^th ^operon, and let *x*_*i *_be the location of the centre of gene *i *in the *l*^th ^operon. To assess the fit of this assignment, *W*_*k*_, the pooled within-operon sum of squares, is used. With  denoting the average location of gene centres in the *l*^th ^operon, *W*_*k *_is defined as(1)

For fixed *k*, an assignment of genes into operons is chosen which minimises *W*_*k*_. The larger a *k *is chosen, the smaller *W*_*k *_will tend to be, but also the explanatory power of the assignment will be reduced. The optimal number of operons is deemed to be the number *k *which minimises the Corrected Akaike Information Criterion (AICC) [44], given by 2(logW_k_) + 2nk/(n-k+1). The additive factor 2nk/(n-k+1) penalises for choosing a large k. The thus predicted operon assignments for each species are given in Additional file 2.

### Shuffled operons

In order to test whether the operon content is informative, we generated shuffled operons. Here the number and size of operons and the number of *che *genes are kept constant, but the *che *genes are shuffled, so that the allocation of *che *genes to operons is randomised.

### Calculating gene functional order from operons

**Figure 2:**
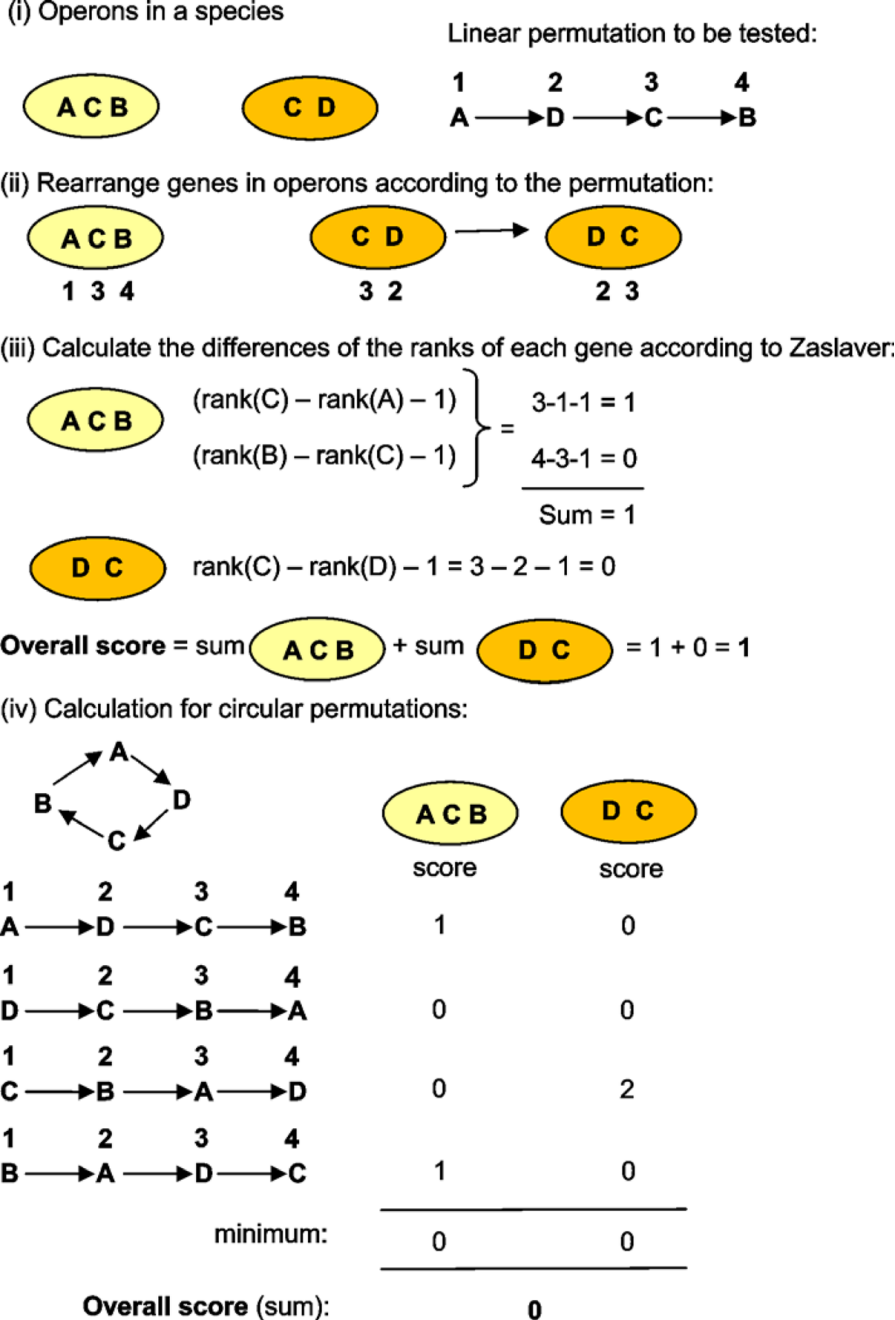
**Flowchart showing the calculation of linear and circular Zaslaver scores**. (i) Given a species with 2 operons containing genes of interest, each linear permutation of gene order can be assessed using the Zaslaver score. (ii) For the gene order A, D, C, B, the genes in the observed operons are first rearranged according to this permutation. (iii) The Zaslaver score is calculated for each operon, then the scores for all operons are summed. For the operon containing genes A, C, B, it can be seen that there is 1 skipped step (gene D) according to the permutation being tested, resulting in a score of 1 for this operon. For the operon containing genes D and C there are no skipped steps, resulting in a score of 0. (iv) The score can be extended to account for circular permutations. Taking each gene in the circular permutation in turn as the starting gene, the linear score for each operon is calculated. The minimum score of these permutations is taken for each operon, and these are then summed to give a final circular Zaslaver score.

The arrangement of genes into operons was also considered, as this has also been shown to be biologically relevant [29,45]. Zaslaver *et al*. [28] developed a scoring scheme to study gene functional order (the order in which gene products are utilised in a linear biological pathway). An example is shown in Figure 2 with details given in Additional file 3 and [28]. For each possible permutation of genes involved in a biological pathway, the genes within operons are arranged according to this permutation. If the operons of a species are strung together and match the given permutation, then the score is 0 for this species. If, however, genes appear in the 'wrong' operon according to the permutation, we then say that functional steps have been skipped. The difference of the rank of each gene is calculated and summed for all species under examination. The lower the score is, the better the gene permutation fits the observed gene arrangements in operons across all the species under investigation. Within operons, it is only the presence or absence of genes, not their order, which affects the Zaslaver score. In most of the pathways studied by Zaslaver, the known functional gene order received the lowest score and therefore had the least number of skipped functional steps, leading to the conjecture that genes within operons tend not to skip functional steps in biological pathways. Zaslaver's score only applies to linear gene orders, which may not be natural for circular biological pathways, as they may contain feedback loops for example. Here, Zaslaver's score is applied to the predicted operons and extended to consider circular gene orders. An example is shown in Figure 2, with more detail given in Additional file 3. Using circular permutations instead of linear permutations, the number of skipped functional steps in an operon is calculated. In contrast to Zaslaver's score, a step is not counted as skipped when the step occurs in a circular organisation of the operon.

### Models of chemotaxis pathways

Parsimonious models for reconstructing chemotaxis pathways are now built by predicting PIs among the multiple Che proteins in a species. In order to build these models, the diversity of patterns of *che *genes in operons is reduced by recording only the types of *che *genes; multiple occurrences of identical *che *genes in the same operon are counted only once.

Among the 1419 operons in the study, 85.7% do not contain multiple occurrences of *che *genes. By far the most frequent multiply occurring gene among the 203 operons with multiple occurrences is *cheY*, with 147 double occurrences, 14 triple occurrences, and 2 quadruple occurrences. Next is *cheW *with 42 double occurrences, then *cheA *and *cheR *both with 3 double occurrences. *cheB *has 1 double occurrence. See Additional file 2 for details.

To model chemotaxis pathways we look at the organisation of *che *genes into operons. PIs between the CheA, B, R, W and Y proteins are predicted using the operon location of the respective genes. In the base model, Model ABRWY, we simultaneously considers 4 types of protein PIs; A~B, A~R, A~W and A~Y. If there is only one CheA homologue in a species, the model assigns PIs between this CheA and all other Che proteins in that species. When there are multiple CheA homologues in a species, we assume that PIs are more likely to be between proteins encoded by genes in the same operon (within-operon) than between proteins encoded by genes from different operons (across operon). We first assign within-operon PIs between CheA and all other Che proteins encoded in the same operon (pseudo-algorithm Step 1). If the resulting pathway is not complete, *i.e*. if it lacks sufficient Che proteins to form a complete set (ABRWY), then cross-talk with proteins encoded in other operons is required (pseudo-algorithm Step 2). In this case incomplete operons which do not yet have edges to other operons are preferred, so that each encoded Che pathway is as distinct as possible. During the reconstruction of pathways, multiple configurations are possible. For example, an incomplete operon may connect to one of several operons having the complementary *che *genes (complementary operons). The preferred configuration is selected based on the operons predicted in all 206 species (Additional file 2), assuming that cross-talk between two operons is more likely if these operons are frequently observed together in many species. Finally, any non CheA-encoding operons are assigned PIs to CheA-encoding operons according to the same frequency principle (pseudo-algorithm Step 3).

### Pseudo-algorithm

#### Step 1: Assigning within-operon PIs

**For **each CheA-encoding operon

Assign within-operon PIs: CheA interacts with all other Che proteins encoded in the operon

#### Step 2: Completing pathways based on CheA-encoding operons

**For **each CheA-encoding operon

**IF **complete pathway **THEN **proceed to next operon

**ELSE **assign across-operon PIs to an operon which is exactly complementary

**IF **complete pathway **THEN **proceed to next operon

**ELSE **assign cross-talks to multiple complementary operons

**IF **complete pathway **THEN **proceed to next operon

**ELSE **assign cross-talks to partner operons which are not CheA-encoding

**IF **complete pathway **THEN **proceed to next operon

**ELSE **assign across-operon PIs to another CheA-encoding operon

#### Step 3: Connecting non CheA-encoding operons

**For **each incomplete operon which does not yet have edges to other operons

Assign PIs to CheA-encoding operon based on the operon co-occurrence frequency across all species

As the distribution of *cheY *in predicted operons is different from the other *che *genes, variants of the above model are devised to explore the possible different PI behaviours of Y. These variant models are abbreviated ABRW+Y, ABRWY+Y, and ABRWY+Y'. Model ABRW+Y considers only three types of PIs, A~B, A~R and A~W in the first step. It follows the same steps to assign protein PIs as the base model, except that Y is excluded; CheY is included in operon prediction, but is ignored when step 3 of the pseudo-algorithm is reached and are connecting operons based on the frequency principle. After the PIs A~B, A~R and A~W are assigned, every Y is connected to every A. The third model, ABRWY+Y, assigns PIs in the same way as the base model, that is by simultaneously considering the four types of PIs A~B, A~R, A~W and A~Y. In addition, the model assigns PIs between every Y and every A in the last step. The two models ABRW+Y and ABRWY+Y therefore propose that a Y can interact with all As. The fourth model, ABRWY+Y', also assigns PIs in the same way as the base model ABRWY in the first step. Then only isolated Ys, *i.e*. Ys not previously connected to any A, are assigned PIs with every A. It is to be emphasised that these models are based on a parsimonious approach, trying to find a simple model which explains a good amount of the observations. There are a number of exceptions from these simple rules.

### Other analyses

Known interaction data for *E. coli *CheY was obtained from the Database of Interacting Proteins (DIP) [46]. Protein disorder was predicted using RONN [47]. T-Coffee [48] and Bl2seq [49] were used for sequence alignment.

## Results and Discussion

206 species were found to have at least one homologue of each of the five core Che proteins (CheA, B, R, W and Y) and 61 (30%) of these have more than one of each, suggesting the existence of multiple chemotaxis pathways in many species.

These 206 species were briefly examined for evidence of flagellar gene homologues to determine whether they are likely to be motile. BLAST searches using the *E. coli *proteins FliC, FliD, FliF, FLiG, FliM, FliN, MotA and MotB were carried out and all hits with an e-value of 10 or less were accepted. 147 species had hits to all of these proteins. 18 species had hits to none of them, but according to HAMAP [50] only 4 of these have no flagella (*Candidatus Methanoregula boonei*, *Desulfococcus oleovorans*, *Thermococcus onnurineus *and *Trichodesmium erythraeum*). However, *Thermococcus onnurineus *is an archaea and KEGG [51] indicates it does have flagella-related proteins present. *Trichodesmium erythraeum *is a cyanobacterium and therefore might move using pili, as may *Candidatus Methanoregula boonei *and *Desulfococcus oleovorans*. We therefore conclude that the 206 species are all likely to be motile rather than using their Che proteins only for other functions. Singh *et al*. [52] also found that the majority of the species in their study which encode genes for CheA, B, R, W and Y were annotated as being motile.

1419 *che *operons were found in the 206 species (Additional file 2) and Table 3 shows the top 10 most frequently observed types. On average, we find that a species has 6.89 (STD 5.05) *che *operons and one operon has 1.98 (STD 1.53) *che *genes. The number of genes in an operon varied between 1 and 9 *che *genes. Table 4 shows the distribution of *che *genes within operons and the number of homologues of each Che protein across the 206 species. Notably, *cheY *is distributed differently from other *che *genes, being frequently found as an isolated *che *gene (*i.e*. with no other *che *genes in close proximity) (Table 2 and Table 3).

**Table 3:** Organisation of *che *genes in the top 10 most frequently found operons

	By operons			By species		
**Rank**	**Operon**	**Number of operons**	**Relative frequency (%)**	**Operon**	**Number of species**	**Relative frequency (%)**

1	Y	708	49.9	Y	170	82.5

2	W	128	9.0	ABRWY	105	51.0

3	ABRWY	121	8.5	W	83	40.3

4	R	85	6.0	R	80	38.8

5	ABWY	63	4.4	ABWY	60	29.1

6	AWY	30	2.1	AWY	28	13.6

7	WY	29	2.0	WY	25	12.1

8	BW	27	1.9	BW	25	12.1

9	AY	26	1.8	ABRY	25	12.1

10	ABRY	25	1.8	AW	24	11.7

A total of 1419 operons were detected in the 206 genomes. 1242 (88%) of these occur in the top 10 operon types.

**Table 4:** Distribution of *che *genes within operons and within species

*che *gene	A	B	R	W	Y
**Number of homologues**	**Number of operons**				

1	329	350	334	441	906

2	3	1	3	42	148

3					14

4					2

Total	332	351	337	483	1070

**Number of homologues**	**Number of species**				

1	125	111	123	72	27

2	51	65	49	57	24

3	18	17	25	33	34

4	8	8	2	21	23

5	2	2	6	8	15

6	2	3	1	4	15

7				2	13

8				6	5

9				1	9

>= 10				2	41

Total	206	206	206	206	206

### Isolated *che *genes, complete operons and complementary operons

**Figure 3:**
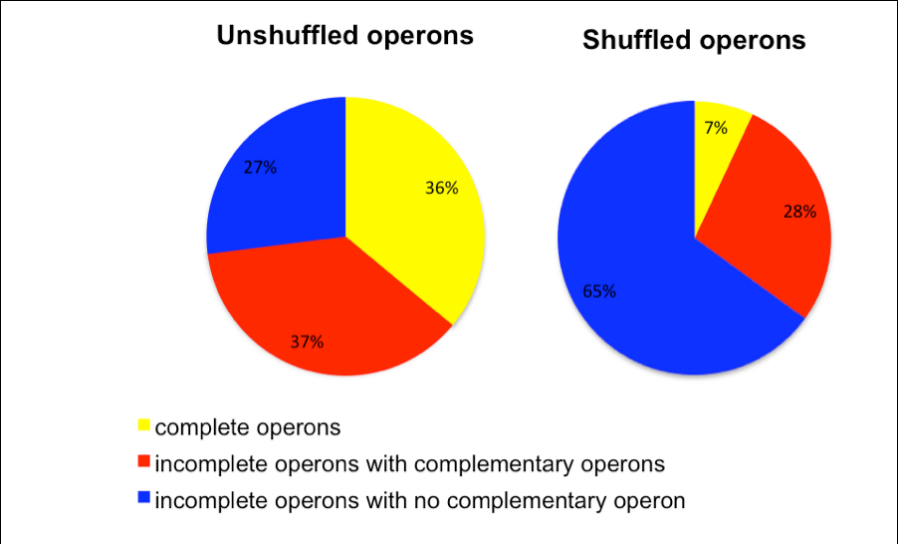
**Comparison of predicted and shuffled operons**. Complete operons contain all 5 essential *che *genes (*A, B, R, W *and *Y*). Incomplete operons are missing one or more of these genes. Complementary operons contain genes which make up a complete set of essential *che *genes. Shuffled operons are generated by keeping the number and size of operons within a species constant, but shuffling the *che *genes so that the allocation of *che *genes into the operons is randomised.

Homologues of *cheA *are very rarely found outside a *che *operon (Table 2). This may be expected as CheA is the point of integration of all chemotaxis receptor signals. Only two species have an isolated *cheA*, but when these are examined in detail, it is found that the *cheA *in *Thiomicrospira crunogena *is adjacent to a *cheD *gene, and in *Opitutus terrae *the *cheA *is close to a both a putative signal transduction protein and a *cheW *transcribed from the opposite DNA strand. They therefore are associated with genes encoding components of chemotaxis pathways. In contrast, *cheY *homologues are commonly found separated from other *che *genes (Tables 2 and 3). More rarely, *cheR *and *cheW *are found outside *che *operons. The benefit of isolating che genes from the main operon is unclear, but may allow independent regulation of expression. The majority of operons in our data are either complete (contain *cheA, B, R, W *and *Y *genes) or have complementary operons (whose combined genes form a complete set) (Figure 3). Among the 101 species which appear to have no complete operons containing all five core *che *genes, 74% have complementary operons, whilst in simulations using shuffled operons only 41% have complementary operons. The distributions of genes in shuffled operons are significantly different from the unshuffled data (p-value < 0.0001, chi-square test of homogeneity). This suggests that the organisation of genes into operons is not arbitrary, and may reflect biological optimisation.

### The order of genes reflects their functional mechanism

**Figure 4:**
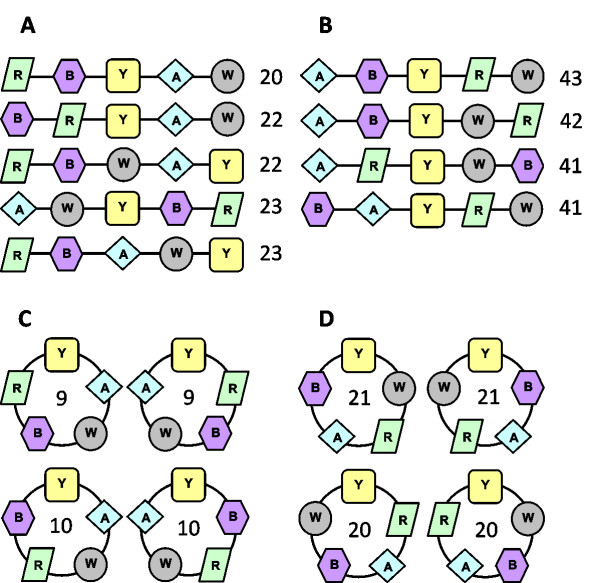
**Permutations of *che *genes for linear and circular gene orders with associated Zaslaver scores**. (A) lowest linear scores (B) highest linear scores (C) lowest circular scores (D) highest circular scores. Note that for linear gene orders, reverse permutations will have the same score. Scores are calculated as described in Figure 2.

The arrangements of genes into operons was investigated by calculating the Zaslaver score for each possible permutation of the *cheA, B, R, W *and *Y *genes, initially assuming that gene order is linear (Figure 2). The lower the score, the better the permutation fits the observed gene arrangements in operons across all species under investigation. Note that any operon containing all 5 *che *genes obtains a score of 0, as there are no skipped functional steps. The results suggest the existence of two organised gene blocks, (*AW*) and (*RB*), which are observed in the permutations with low scores but not in those with high scores (Figure 4A and 4B). In contrast, *cheY*s are found to be in any position outside these blocks. This implies a circular gene order (AW)(RB)Y, which is reasonable given the underlying circular pathway. We therefore developed an extension of the Zaslaver score for investigating circular gene orders. The two circular orders with the lowest scores also contain the A~W and R~B blocks, whereas the two with the highest scores do not (Figure 4C and 4D). These results imply that operons are not split randomly and that there is a preference for the pairs (*AW*) and (*RB*) to be together within operons. Additional file 4 gives details of the most frequently observed operons in our data set and their associated Zaslaver scores.

The Zaslaver scores imply that A~W and B~R occur *together *in operons but do not show us that they are *adjacent *in operons. We therefore went on to analyse the physical order of genes within operons by calculating the relative frequencies of pairs of consecutive genes. The results show that *cheA *and *cheW *do tend to be adjacent in operons, as do *cheR *and *cheB *(Table 5); AW appears 176 times and RB appears 165 times. There also seems to be a clearly preferred order of genes, with A generally occurring before W and R before B.

**Table 5:** Relative occurrence of ordered gene pairs

		2^**nd **^gene					
	**Raw occurrence**	**A**	**B**	**R**	**W**	**Y**	**Sum**

	A	0	54	29	176	34	293

	B	34	1	34	45	84	198

1^*st *^gene	R	6	165	2	7	31	211

	W	27	47	130	27	40	271

	Y	191	42	14	48	117	412

							1385

		**2^**nd **^gene**					

	**Relative occurrence**	**A**	**B**	**R**	**W**	**Y**	**Sum**

	A	0.00	0.18	0.10	**0.60**	0.12	1

	B	0.17	0.01	0.17	0.23	0.42	1

1^*st *^gene	R	0.03	**0.78**	0.01	0.03	0.15	1

	W	0.10	0.17	0.48	0.10	0.15	1

	Y	0.46	0.10	0.03	0.12	0.28	1

							5

The most frequently occurring ordered gene pairs (R followed by B and A followed by W) are shown in bold.

Close proximity of genes may indicate that the proteins they encode are co-localised to form a complex, aided by co-transcription and co-translation. Dandekar et al. found that conservation of gene order was a 'fingerprint' of proteins which physically interact [45]. CheA and CheW have been shown to interact *in vitro *and *in vivo *[53,54], and seen to interact in a crystal structure (PDB identifier 2CH4[55]). It has also been shown that CheA and CheW co-localise to the MCPs in *E. coli *[56,57]. However, no interaction between CheR and CheB has been shown. While CheR and CheB co-localise to the transmembrane receptors in *E. coli *[5,58], in *R. sphaeroides*, CheR2 and R3 are localised to the cytoplasmic and membrane bound chemosensory clusters respectively. However, CheB1 and B2 are diffuse in the cytoplasm, making the role of localisation unclear.

### Comparison to known chemotaxis operons

51% of the 206 species studied and 82% of the 61 species with putative multiple pathways have at least one complete chemotaxis operon (containing *cheA, B, R, W *and *Y*). In order to ascertain if these operons may encode similar pathways to those identified in *R. sphaeroides*, we examined whether known non *che *genes are also found within the operons. The proteins encoded in *R. sphaeroides *CheOp3 localise in cytoplasmic clusters with Tlp receptors. PpfA, encoded in CheOp3, is known to be critical for correct partitioning of these protein clusters upon cell division [16]. However, PpfA is a ParA homologue, and ParA-ParB pairs may be involved in DNA segregation upon cell division [59]. We found that 77 species (37%) have a PpfA homologue encoded in a *che *operon *without *a ParB homologue being encoded in the same operon (Additional file 5). 86% of these species also have a putative cytoplasmic chemotaxis receptor homologue. Strikingly, 61% of species that potentially have multiple chemotaxis pathways have a PpfA homologue encoded in a *che *operon, and of these 97% also have a putative cytoplasmic chemotaxis receptor homologue. The fact that separate groups of *che *genes are found so often on bacterial genomes and that *ppfA*, a non *che *gene, is often present in these operons suggests that not only is the grouping of *che *genes a common way of separating chemotaxis pathways but that organised cytoplasmic clusters of Che proteins may be present in a significant number of species.

### Prediction of chemotaxis pathways

We predict chemotaxis pathways in all 206 species using the 4 models described in the methods. When building these models, we ignore multiple occurrences of *che *genes in an operon, as we assume that each copy of a *che *gene in an operon will have the same interaction behaviour. Of the 1419 operons analysed, 203 (14%) have multiple occurrences of one or more *che *genes. The most frequently observed *che *gene to have multiple copies within a *che *operon is *cheY*, with multiple copies occurring in 164 operons (Additional file 6). Our 4 models explicitly consider different possible PI behaviours of cheY. The other four *che *genes, *A, B, R *and *W*, occur as multiple copies in only 3, 1, 3 and 42 operons respectively.

We calculate the occurrences of each type of PI (Additional file 7) for all 4 models. Due to the fact that multiple copies of *che *genes in operons are ignored, recording an occurrence is to be understood as "there is at least one PI of this type" taking place. For example, in the operon AWW we count one within-operon A~W PI. With this interpretation, our conclusions are not affected by multiple occurrences of a gene within an operon. As Che proteins may take on multiple functional roles, it is to be noted that ignoring multiplicity of copies may have resulted in neglecting some interesting phenomena. This decision was reached not only for the sake of parsimony, but also due to the lack of information about these multiple roles and their effects on chemotaxis pathways. We also do not explicitly consider the multiple domain organization of proteins in our models. In the case of the five-domain protein CheA, we put this protein in a unique position such that all other proteins interact with it directly or indirectly. As a multiple domain protein, CheA has an central role in our models (Figure 1). It has PIs to CheW, CheY, CheB and CheR, which may reflect the connectivity from its multiple domains.

The chi-square tests of homogeneity (Additional file 8) show that models ABRWY, ABRWY+Y and ABRWY+Y' have similar PI patterns for A~B, A~R and A~W, whereas these PIs in model ABRW+Y are significantly different from the other models. When the A~Y PI is included, all models are significantly different, except ABRWY and ABRWY+Y'. The PI behaviour of CheY is still not fully understood, so all three possible PI behaviours of CheY discussed here are plausible.

### PI behaviour of Che proteins

The predicted PIs from each model are then compared to those derived using shuffled operons (Additional file 9 and 10). In all 4 models the predicted PIs are significantly different from shuffled PIs (all p-values < 0.0001, chi-square tests of homogeneity) suggesting again that the organisation of *che *genes into operons is not arbitrary.

In all 4 models, the PI between A and W ranks first among within-operon PIs, whereas it is always the last among across-operon PIs (Table 6). The PIs between A and W tend to occur within-operon rather than across-operon, possibly due to the signal-transduction complex formed by receptor-CheA-CheW, and in order to simplify the control mechanism.

**Table 6:** Ranking of the relative occurrence of PIs in predicted pathways

	Model			
**PI**	**ABRWY**	**ABRW+Y**	**ABRWY+Y**	**ABRWY+Y'**

A~W*				

A~B*				

	A~Y*	A~Y**	A~Y**	A~Y*

		A~Y*	A~Y*	

A~R*				

	A~Y**			

A~R**				

A~B**				

				A~Y**

A~W**				

* Within-operon PIs; ** Across-operon PIs

These rankings are based on the difference between the relative occurrence of PIs from predicted pathways and the relative occurrence of PIs from shuffled pathways (Additional file 8). Relative occurrences are calculated as (number of observed occurrences of PIs of type A~X)/(total number of observed occurrences of PIs).

**Figure 5:**
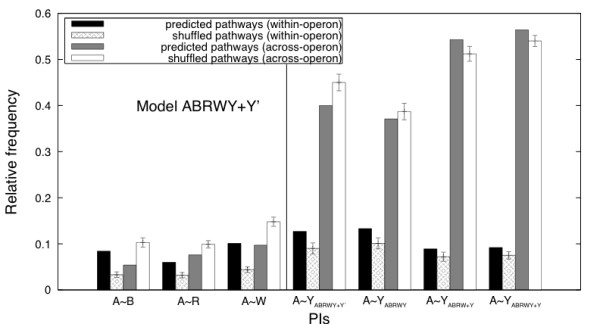
**The relative occurrences of PIs in the predicted pathways and shuffled pathways**. The relative occurrence of PIs are calculated from the predicted pathways based on our models and from the 100 simulations of shuffled pathways. The error bars are calculated by (2* standard error). The standard error is calculated from 100 simulations of shuffled pathways. Relative occurrences are calculated as (number of observed occurrences of PIs of type A~X)/(total number of observed occurrences of PIs of all types).

Although the occurrence of within-operon A~Y PIs for all models is significantly higher than in shuffled pathways, the occurrence of across-operon A~Y PIs is model dependent (Figure 5). In contrast to the other models, in model ABRWY (where Y is treated in an identical way to A, B, R and W), the A~Y across-operon occurrence isnot significantly different to that in shuffled pathways. This suggests that the A~Y PI behaves differently to the other PIs and that the other models, in which Y is treated differently to other Che proteins, may be more appropriate.

Model ABRWY+Y' is the most parsimonious of our models satisfying our assumptions. The ranking of the relative frequencies of PIs shows that all within-operon PIs rank higher than cross-operon PIs (Table 6 and Additional file 10), unlike for models ABRW+Y and model ABRWY+Y. Figure 5 compares the relative occurrence of PIs in the predicted pathways to their relative occurrence in randomly shuffled pathways, where the *che *genes are assigned to operons at random. For all PIs, the within-operon relative occurrence in the predicted pathways are significantly higher than the occurrence using shuffled pathways. For A~B, A~R and A~W, the across-operon relative occurrence of PIs in the predicted pathways are significantly lower than in randomly shuffled pathways. This finding is consistent with our model assumption that within-operon PIs are used in preference to across-operon PIs. In contrast, for A~Y, whether the across-operon PIs yield a higher or lower relative occurrence compared to randomly shuffled pathways is model dependent. In model ABRWY there is no significant difference. In model ABRWY+Y', across-operon occurrence of PIs in the predicted pathways are rarer than in shuffled pathways, but in models ABRW+Y and ABRWY+Y, the predicted pathways result in more occurrence of across-operon PIs than shuffled pathways. This finding suggests that the A~Y PI behaves differently to the other PIs, and the models in which Y is treated differently to other Che proteins may be more appropriate than Model ABRWY. For all models, across-operon A~Y PIs occur with much higher frequency than A~B, A~R and A~W across-operon PIs. This supports our finding that isolated CheYs, though not different in sequence to those encoded within *che *operons, may differ in their expression patterns, and hence their ability to interact *in vivo*.

### CheY may have additional functions

The CheY-like receiver domain (REC domain) is a common regulatory module in many bacterial proteins [60]. It is frequently found in association with DNA-binding domains but is also found as a domain in other proteins, such as in CheB, and can function alone as anSDRR. Distinguishing *cheY *genes from those encoding non-CheY SDRRs has so far proved impossible from sequence alone, hence the high frequency of isolated '*cheY*' genes may be spurious. However, it may also imply that CheY has additional functions and interacts with proteins other than those in chemotaxis pathways. Further evidence for this comes from the 50 species found which have at least 1 CheY homologue but no other Che proteins (A, B, R, W, V or Z), and from the Database of Interacting Proteins (DIP) which suggests that *E. coli *CheY interacts with the pyruvate dehydrogenase complex (PDHc). PDHc ultimately causes formation of acetyl-CoA, and thus possibly CheY can autoacetylate with acetyl-CoA as the acetyl donor; it is known that acetylation of CheY can activate it and can generate clockwise flagellar rotation [61]. In addition, CheY6 from *R. sphaeroides*, discussed shortly, may be a paradigm for a specific type of CheY which may be capable of binding to multiple ligands.

### Comparison of predicted PIs to interactions reported in the literature

We compare our predicted pathways to those interactions reported in the literature. The prediction for *E. coli *is straightforward as the pathway only involves one operon, and our predictions, assigning all within-operon PIs, are verified [35] (Additional file 11). Similarly, the predictions are verified for other organisms with only one complete operon, such as *Salmonella enterica *serovar Typhimurium and *Sinorhizobium meliloti *[62].

*P. aeruginosa *has 5 operons containing homologues of *E. coli *chemotaxis proteins, designated as Clusters I-V. Our four models correctly predict all known PIs, including the across-operon PI between A (Cluster I) and R (Cluster V) (Additional file 12). The models also predict unreported PIs in Cluster II. The predicted pathways coincide with the previous finding that Cluster II functions separately from Cluster I [32].

**Figure 6:**
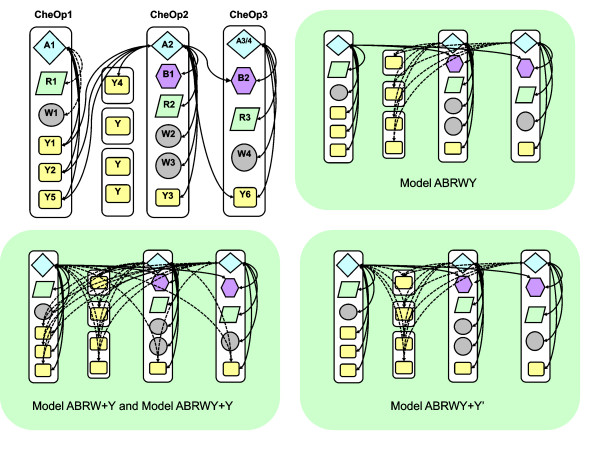
**Predicted pathways for *R. sphaeroides***. The known *in vitro *phosphorylation reactions are shown in the top left. Dotted lines between A1 and R1 and A1 and W1 are assumed, but not experimentally verified, interactions. The predicted pathways for our different models are shown with a green background. Models ABRW+Y and ABRWY+Y give the same prediction. Dotted lines here indicate predicted across-operon PIs. The 3 CheYs shown with no numbers were found by our BLAST searches but are not annotated as CheYs in the literature.

In *R. sphaeroides *most of the chemotaxis genes are found in three operons, CheOp1, CheOp2 and CheOp3. There is limited interaction data available for CheOp1 as it is not expressed under laboratory conditions, although it has been shown that CheA1 can phosphorylate CheY1, CheY2 and CheY5, but not CheB1 or CheB2 *in vitro *[21]. However, much more experimental interaction data are available for CheOp2 and CheOp3, and *in vitro *cross-talk between CheOp2 and CheOp3 has been reported [8,63]. Models ABRWY and ABRWY+Y' do not predict the PI between A2 (CheOp2) and Y6 (CheOp3), as the two pathways are already complete (Figure 6). This particular PI apparently contradicts the assumption that the pathways tend to be distinct, suggesting different PI behaviour between A and Y compared to other Che proteins [64]. However, *in vivo *the pathways are physically separate and the pathways therefore are probably physiologically distinct [10]. In our models ABRW+Y and ABRWY+Y, Ys encoded by isolated genes are predicted to have PIs with the proteins encoded in both CheOp2 and CheOp3.

### Identifying pathways in *R. sphaeroides *using sequence information

As a potential method to improve our models, we examined the Che homologues in *R. sphaeroides *to see if it was possible to identify to which pathway a Che homologue would belong based on sequence level properties. However, this proved extremely difficult. For example CheW2 and CheW3, which are present in the same pathway, are no more similar to each other than to the other CheW homologues (Additional file 13). Even residues thought to be involved in contacts to CheA are not conserved between homologues from the same gene group. Further examination using the Evolutionary Trace method [65], where both sequence and structure are considered, also revealed no patterns of conservation. In addition, regions of the membrane-bound and cytoplasmic chemotaxis receptors putatively used for binding to CheA/CheW [55] are found to be extremely similar (Additional file 14), suggesting that both these types of receptors could bind to all the CheA/CheW homologues *in vitro*. It is also known that CheY5, encoded by CheOp1, can restore chemotaxis in a CheY3/CheY4 deletion mutant when expressed from a plasmid (unpublished data - JPA), and CheA2 has been shown to phosphorylate all CheY homologues in *R. sphaeroides in vitro *[21]. Given this evidence, we propose that localization of proteins into distinct clusters in the cell, based on their operon groupings, is likely to be the key determinant separating pathways *in vivo*.

### The Non-classical CheA homologues of *R. sphaeroides *are rare

CheA in *E. coli *is made up of 5 domains (P1 to P5). In *R. sphaeroides*, CheA3 only has two of these domains, P1 and P5, connected by a long linker. This architecture was not found repeated in any of the complete genomes searched, and an online search of CDART [39] revealed a similar protein only in the related *Roseovarius sp., Caldicellulosiruptor saccharolyticus, Desulfuromonas acetoxidans *and *Anaerocellum thermophillum*. However, the linker region between the two domains is considerably shorter than that in CheA3 for all the but the protein in *Roseovarius sp*. In *R. sphaeroides *there is a second non-classical CheA, CheA4, which has only P3, P4 and P5 domains. Only 4 other species examined had such a CheA homologue (*Caldicellulosiruptor saccharolyticus, Thermoanaerobacter tengcongensis, Salinibacter ruber *and *Agrobacterium vitis*). These non-classical CheA homologues are therefore apparently very rare, and as such are probably not a useful paradigm for modelling, although sequencing of more bacterial genomes may reveal other such proteins in the future.

### CheY6 from *R. sphaeroides*

**Figure 7:**
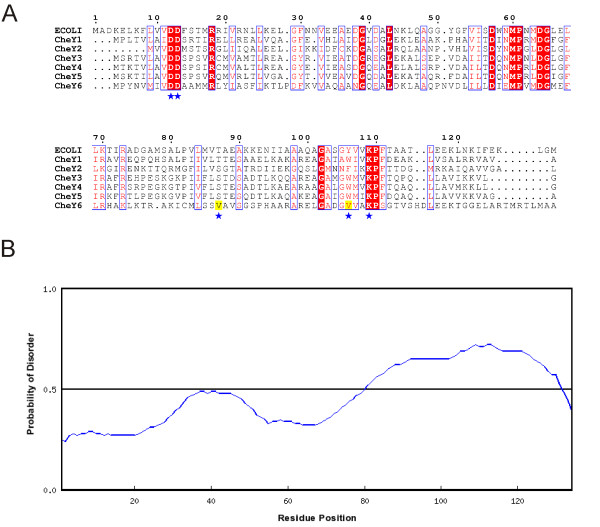
**Differences between CheY6 and classical CheY proteins**. (A) Alignment of *R. sphaeroides *CheY1-6 and *E. coli *CheY. Blue stars: residues essential for phosphorylation of D57 in E. coli (D12, D13, T87, Y106 and K109). Residues highlighted in yellow in CheY6 show important differences to other CheY homologues. This figure was made using ESPript [66]. (B) RONN [47] disorder prediction for *R. sphaeroides *CheY6 showing the disordered C-terminus.

CheY6 from *R. sphaeroides *differs significantly in sequence from *E. coli*-type CheY homologues (Figure 7A) and is in fact more similar in sequence to the REC domain of CheB proteins. When CheY6 was used as a BLAST query sequence against the full database of 523 species, 72 new CheY homologues were found in 49 different species, some of which were encoded in *che *gene groups (Additional file 15). 45 of these species also encode a classical CheY homologue, suggesting CheY6 alone may not be sufficient for chemotaxis and a classical CheY homologue must also be present.

The aromatic residue Y106 is known to be involved in *E. coli *CheY function. However, CheY6 lacks an equivalent residue (Figure 7A), yet it is the only CheY homologue in *R. sphaeroides *which is able to stop the flagellar motor [64]. We also predict that CheY6 has a disordered C-terminal region not seen in the other CheY homologues (Figure 7B). The combination of these differences may help to explain why CheY6 is found to auto-dephosphorylate ten times faster than CheY1-5. The flexible, disordered region may allow CheY6 to bind to multiple ligands. In order to ascertain whether there are CheY6-like homologues in other species, the extent of disorder in CheY homologues across all species was examined. 103 species (50% of all species studied) were found to have a CheY homologue with significant disorder present at the C-terminus. Of the 207 CheY homologues with C-terminal disorder (Additional file 16), 118 (from 77 species) were found in a *che *operon, as is CheY6 from *R. sphaeroides*, and 89 (from 60 species) were isolated. 37 species have a CheY homologue which has C-terminal disorder and is also lacking the important aromatic residue. 52 of the 61 species with more than one of each Che protein (85%) have a CheY homologue with C-terminal disorder, and 19 of these species have a CheY-homologue which is also lacking the aromatic residue. This suggests that CheY6 may be a common type of CheY, but must be identified by the presence of a disordered C-terminus combined with the lack of aromatic residue, rather than by sequence searches alone.

## Conclusions

Bacterial chemotaxis is widely used in systems biology as a paradigm for signal processing. If this system can be fully understood, it would provide a basis for understanding other, more complex signalling systems. Chemotaxis in *E. coli*, and a few other species such as *R. sphaeroides*, has been widely studied, but the extent to which chemotaxis pathways in these species are representative of bacterial chemotaxis as a whole has not yet been established. This work aims to address this by undertaking an analysis of a large, non-redundant set of complete bacterial genomes.

We show that homologues of all of the core chemotaxis proteins (CheA, B, R, W and Y) are present in many species. We developed a novel operon identifier and show that *che *genes tend to be grouped into putative operons, with complete operons containing all 5 *che *genes being found in 51% of species examined.

The existence of multiple homologues of all these proteins in 30% of the 206 species studied suggests that the presence of more than one chemotaxis pathway is relatively common, and therefore that the *E. coli *paradigm of chemotaxis is not appropriate for a large number of bacteria. The question then arises as to how the different chemotaxis pathways in such species are kept distinct. In *R. sphaeroides*, proteins involved in two different chemotaxis pathways are known to be expressed simultaneously from two separate operons, but the proteins of one pathway are localised to the cell poles and the proteins of the other to a cluster in the cytoplasm. The PpfA protein, encoded in CheOp3, is known to be critical for the correct partitioning of the cytoplasmic Che protein cluster on cell division. We found PpfA homologues within our *che *operons in 37% of species studied, and in 61% of species which putatively have multiple chemotaxis pathways. This suggests that cytoplasmic clusters of Che proteins may occur in many other bacteria. We propose that grouping of *che *genes into operons and localization of proteins into clusters in the cell are likely to be the major factors determining the separation of multiple chemotaxis pathways within a species. Chemotaxis in *R. sphaeroides *may therefore provide a useful model for species with multiple chemotaxis pathways. However, this species encodes some apparently non-typical chemotaxis components, CheA3, CheA4 and CheY6. We found that the variants of CheA proteins are rare in the species we examined. However, the CheY6 variant appears to be a common type of CheY, with a significantly disordered C-terminal region which may be functionally significant

The grouping of chemotaxis genes from a large number of species into putative operons allowed us to examine the general distribution of *che *genes in bacteria. While most *che *genes, particularly *cheA*, were usually found to be situated within *che *operons, the distribution of *cheY *is different, with isolated *cheY *genes being extremely common. CheY PI behaviour was also predicted to be different to that of other Che proteins, and models which take these factors into account are likely to be more realistic than those which treat all Che proteins in an identical way.

Finally, gene order in *che *operons was found to be important with *cheA-cheW *and *cheR-cheB *blocks observed in our data. These likely reflect functional linkage of the encoded proteins. In general, the organisation of genes into operons may provide information for the inference of gene functional order, and conserved proximity between genes may suggest that the genes are involved in similar biological mechanisms. The order of genes appears to be important at both the within-operon and between-operon levels.

## Authors' contributions

RH collected the data, carried out the BLAST searches and bioinformatics analyses and drafted the manuscript. PYC carried out the gene clustering and modelling and helped to draft the manuscript. JPA participated in the design of the study. GR and CMD participated in the design and coordination of the study and helped to draft the manuscript. All authors read and approved the final manuscript.

## Supplementary Material

Additional file 1 **Table S1 Location of *che *genes for all species studied**. Spreadsheet giving the accession numbers and gene locations for all *che *genes found in 474 bacterial species with a BLAST hit to at least one Che protein. Click here for file

Additional file 2 **Table S2 Predicted operons for all species studied**. Spreadsheet giving the operons predicted for each of the 474 species with a BLAST hit to at least one Che protein. Click here for file

Additional file 3 **Text S1 Explanation of the score from Zaslaver *et al***. Mathematical explanation of the calculation of the score from Zaslaver *et al*. for lineargene orders and the calculation for circular gene orders. Click here for file

Additional file 4 **Table S3 The most frequently observed operons in our data set and their associated Zaslaver scores**. Click here for file

Additional file 5 **Text S2 Species with PpfA homologues**. List of the 77 species with a PpfA homologue in a che operon. Click here for file

Additional file 6 **Table S4 Operons used in the study**. Table showing all 1419 operons and their compressed equivalents, where multiple copies of che genes have been ignored. Click here for file

Additional file 7 **Table S5 Frequency of PIs in predicted pathways**. Table showing the frequencies, relative frequencies and overall relative frequencies of within- and across-operon PIs for predicted pathways using the 4 models. Click here for file

Additional file 8 **Table S6 P-values in chi-square tests of homogeneity**. Table showing the p-values for testing differences between models when considering PIs A~B, A~R and A~W alone, then considering A~Y as well, using a chi-square test of homogeneity. Click here for file

Additional file 9 **Table S7 Occurrence of PIs in shuffled pathways**. Table showing the occurrences, relative occurrences and overall relative occurrences of within- and across-operon PIs for pathways constructed using shuffled operons using the 4 models. Click here for file

Additional file 10 **Table S8 Ranking of PIs**. Table showing the unshuffled and shuffled relative occurrences for each PI, their difference, and the final ranking based on this difference. Click here for file

Additional file 11 **Figure S1 Chemotaxis pathway of *E. coli***. Known and predicted core chemotaxis pathways in *E. coli* Click here for file

Additional file 12 **Figure S2 Chemotaxis pathways of *Pseudomonas aeruginosa***. Known and predicted chemotaxis pathways in *Pseudomonas aeruginosa* Click here for file

Additional file 13 **Table S9 Statistics for *R. sphaeroides *CheW alignments**. Table showing the alignment statistics derived from bl2seq for alignment of each of the 4 CheW homologues in *R. sphaeroides *and for each of these 4 homologues aligned to *E. coli *CheW. Click here for file

Additional file 14 **Figure S3 Alignment of CheA/CheW binding region of MCPs and TLPs**. Alignment of the region at the tip of the all 9 MCPs, tlpL and tlpT from *R. sphaeroides*. Click here for file

Additional file 15 **Text S3 Sequences of CheY homologues**. Sequences in fasta format of the 72 CheY homologues which were only found when using CheY6 from *R. sphaeroides *as a BLAST query sequence. Click here for file

Additional file 16 **Text S4 Sequences of CheY homologues with C-terminal disorder**. Sequences in fasta format of the 207 CheY homologues which have C-terminal disorder. Click here for file
