# Supplementary material for: Deciphering chemotaxis pathways using cross species comparisons
Source: BMC Syst Biol. 2010 Jan 11;4:3. doi: 10.1186/1752-0509-4-3 (PMC2829493; doi:10.1186/1752-0509-4-3)
Supplement: Additional file 3 — Text S1 Explanation of the score from Zaslaver et al. Mathematical explanation of the calculation of the score from Zaslaver et al. for lineargene orders and the calculation for circular gene orders. [file 1752-0509-4-3-S3.PDF]

Text S1. Explanation of the score from Zaslaver *et al.* [1]

Score for linear pathways

Let $\{y_1, y_2, \cdots, y_n\}$ be a set of n genes, and let $(y_{p(1)}, y_{p(2)}, \cdots, y_{p(n)})$ be a permutation of $\{y_1, y_2, \cdots, y_n\}$. In a species *s* with *k* operons, consider a given operon *l* ($l \in \{1,2,\cdots,k\}$) of n_l genes, $x_1, x_2, \cdots, x_{n_l}$. Let $x'_1, y'_2, \cdots, x'_{n_l}$ be the ordered list of these n_l genes according to order given in *P*, so x'_1 is the y_j which comes first in the ordered sequence $y_{p(1)}, y_{p(2)}, \cdots, y_{p(n)}$ among the genes in the operon, x'_2 is the y_j which comes second, and so on. Note that x'_i does not have to equal $y_{p(i)}$.

The Zaslaver score counts the number of skipped steps of *P* in all operons:

$$\text{Zaslaver score} = Z(P) = \sum_s \sum_{l=1}^{k} \sum_{j=2}^{n_l} \left[R(x'_j; P) - R(x'_{j-1}; P) - 1 \right]$$

where $R(x'_j; P)$ is the rank of x'_j in *P*.

Score for circular pathways

Assume that $\{y_1, y_2, \cdots, y_n\}$ is a set of genes of interest. Let $(y_{p(1)}, y_{p(2)}, \cdots, y_{p(n)})$ be a fixed permutation of $\{y_1, y_2, \cdots, y_n\}$. A circular permutation is an arrangement of objects around a fixed circle, so for example (A, B, C, D) and (B, C, D, A) describe the same circular permutation, whereas (A, C, B, D) describes a different circular permutation. Let *A* be one observed arrangement of *m* operons $\{a_1, a_2, \cdots, a_m\}$ and let $\{x_{l,1}, x_{l,2}, \cdots, x_{l,n_l}\}$ be the genes within the operon a_l. Denote by Q_i^P the representation of the circular permutation to which *P* belongs, written as starting at the ith position in *P*. We scan all species to construct a collection Z of all observed arrangements of operons, each arrangement being considered only once.

Let $R(x_{l,j}; Q_i^P)$ be the rank of the jth gene in a_l according to Q_i^P. The number of skipped steps in *A*, given the permutation Q_i^P, is as follows:

$$C(Q_i^P, A) = \sum_{l=1}^{m} \sum_{j=2}^{n_l} \left[R(x_{l,j}; Q_i^P) - R(x_{l,j-1}; Q_i^P) - 1 \right].$$

There is no score for singleton operons. Finally, the score for the circular permutation to which P belongs being observed in *Z* is the summation of the minimal subscores from all arrangements of operons:

$$S(P, Z) = \sum_{A \in Z} \min\left\{ C(Q_i^P, A), i = 1, \cdots, n \right\}.$$

1. Zaslaver A, Mayo A, Ronen M, Alon U: **Optimal gene partition into operons correlates with gene functional order**. *Physical Biology* 2006, **3**(3):183-189.
