# Supplementary material for: Deciphering chemotaxis pathways using cross species comparisons
Source: BMC Syst Biol. 2010 Jan 11;4:3. doi: 10.1186/1752-0509-4-3 (PMC2829493; doi:10.1186/1752-0509-4-3)
Supplement: Additional file 7 — Table S5 Frequency of PIs in predicted pathways. Table showing the frequencies, relative frequencies and overall relative frequencies of within- and across-operon PIs for predicted pathways using the 4 models. [file 1752-0509-4-3-S7.PDF]

Table S5 Frequency table of the PIs in predicted pathways

Model ABRWY	Within operon			Across operon			
	Freq.	Rel. freq.	Overall Rel. freq.	Freq.	Rel. freq.	Overall Rel. freq.	
A~B	245	0.226	0.089	157	0.093	0.057	
A~R	175	0.162	0.063	221	0.131	0.080	
A~W	293	0.271	0.106	281	0.167	0.102	
A~Y	369	0.341	0.133	1026	0.609	0.371	
Total	1082	1		1685	1		2767
Model ABRW+Y	Within operon			Across operon			
	Freq.	Rel. freq.	Overall Rel. freq.	Freq.	Rel. freq.	Overall Rel. freq.	
A~B	245	0.226	0.059	199	0.065	0.048	
A~R	175	0.162	0.042	251	0.082	0.061	
A~W	293	0.271	0.071	359	0.117	0.087	
A~Y	369	0.341	0.089	2249	0.735	0.543	
Total	1082	1		3058	1		4140
Model ABRWY+Y	Within operon			Across operon			
	Freq.	Rel. freq.	Overall Rel. freq.	Freq.	Rel. freq.	Overall Rel. freq.	
A~B	245	0.226	0.061	157	0.054	0.039	
A~R	175	0.162	0.044	221	0.076	0.055	
A~W	293	0.271	0.073	281	0.097	0.070	
A~Y	369	0.341	0.092	2249	0.773	0.564	
Total	1082	1		2908	1		3990
Model ABRWY+Y’	Within operon			Across operon			
	Freq.	Rel. freq.	Overall Rel. freq.	Freq.	Rel. freq.	Overall Rel. freq.	
A~B	245	0.226	0.084	157	0.086	0.054	
A~R	175	0.162	0.060	221	0.122	0.076	
A~W	293	0.271	0.101	281	0.154	0.097	
A~Y	369	0.341	0.127	1160	0.638	0.400	
Total	1082	1		1819	1		2901
