# Supplementary material for: Deciphering chemotaxis pathways using cross species comparisons
Source: BMC Syst Biol. 2010 Jan 11;4:3. doi: 10.1186/1752-0509-4-3 (PMC2829493; doi:10.1186/1752-0509-4-3)
Supplement: Additional file 8 — Table S6 P-values in chi-square tests of homogeneity. Table showing the p-values for testing differences between models when considering PIs A~B, A~R and A~W alone, then considering A~Y as well, using a chi-square test of homogeneity. [file 1752-0509-4-3-S8.PDF]

Table S6. *P*-values in chi-square tests of homogeneity for models

Model	ABRWY	ABRW+Y	ABRWY+Y	ABRWY+Y’
Considering PIs A~B, A~R and A~W				
ABRWY		0.006265	1	1
ABRW+Y			0.002347	0.002347
ABRWY+Y				1
ABRWY+Y’				
Considering PIs A~B, A~R, A~W and A~Y				
ABRWY		<0.0001	<0.0001	0.2038
ABRW+Y			<0.0001	<0.0001
ABRWY+Y				<0.0001
ABRWY+Y’				
