# Supplementary material for: Deciphering chemotaxis pathways using cross species comparisons
Source: BMC Syst Biol. 2010 Jan 11;4:3. doi: 10.1186/1752-0509-4-3 (PMC2829493; doi:10.1186/1752-0509-4-3)
Supplement: Additional file 9 — Table S7 Occurrence of PIs in shuffled pathways. Table showing the occurrences, relative occurrences and overall relative occurrences of within- and across-operon PIs for pathways constructed using shuffled operons using the 4 models. [file 1752-0509-4-3-S9.PDF]

Table S7. Frequency table of the PIs in shuffled pathways

Model ABRWY	Within operon					Across operon					
	Avg. Freq.	Col. Rel. freq.	std	Rel. freq.	std	Avg. Freq.	Col. Rel. freq.	std	Rel. freq.	std	
A~B	117	0.164	0.01082	0.037	0.0026	368	0.148	0.00576	0.115	0.00454	
A~R	115	0.161	0.01224	0.036	0.0031	351	0.142	0.00553	0.11	0.00444	
A~W	160	0.224	0.01371	0.05	0.00351	523	0.211	0.00664	0.164	0.00519	
A~Y	321	0.45	0.01476	0.101	0.00559	1238	0.499	0.00827	0.387	0.00851	
Total	713	1.000				2481	1.000				3195
Model ABRW+Y	Within operon					Across operon					
	Avg. Freq.	Col. Rel. freq.	std	Rel. freq.	std	Avg. Freq.	Col. Rel. freq.	std	Rel. freq.	std	
A~B	118	0.167	0.01267	0.027	0.00226	417	0.111	0.00501	0.093	0.00451	
A~R	115	0.162	0.01307	0.026	0.00218	408	0.108	0.00486	0.091	0.00425	
A~W	156	0.219	0.01298	0.035	0.00244	646	0.172	0.00691	0.144	0.00621	
A~Y	322	0.452	0.01882	0.072	0.00469	2295	0.609	0.00995	0.512	0.00763	
Total	712	1.000				3768	1.000				4480
Model ABRWY+Y	Within operon					Across operon					
	Avg. Freq.	Col. Rel. freq.	std	Rel. freq.	std	Avg. Freq.	Col. Rel. freq.	std	Rel. freq.	std	
A~B	119	0.167	0.01166	0.028	0.00232	366	0.103	0.00419	0.086	0.00372	
A~R	112	0.159	0.01166	0.027	0.00223	355	0.1	0.00415	0.084	0.00366	
A~W	159	0.224	0.01437	0.038	0.00287	523	0.148	0.0053	0.123	0.00487	
A~Y	319	0.45	0.01718	0.075	0.0039	2298	0.649	0.00817	0.54	0.00601	
Total	711	1.000				3544	1.000				4255
Model ABRWY+Y’	Within operon					Across operon					
	Avg. Freq.	Col. Rel. freq.	std	Rel. freq.	std	Avg. Freq.	Col. Rel. freq.	std	Rel. freq.	std	
A~B	117	0.165	0.01341	0.033	0.00288	367	0.129	0.00578	0.103	0.00461	
A~R	115	0.162	0.01148	0.032	0.0026	351	0.124	0.00423	0.099	0.00354	
A~W	157	0.221	0.01339	0.044	0.00328	525	0.185	0.00594	0.148	0.00501	
A~Y	321	0.452	0.01783	0.09	0.00559	1600	0.563	0.00866	0.45	0.00874	
Total	710					2844					3555
