# Supplementary material for: Deciphering chemotaxis pathways using cross species comparisons
Source: BMC Syst Biol. 2010 Jan 11;4:3. doi: 10.1186/1752-0509-4-3 (PMC2829493; doi:10.1186/1752-0509-4-3)
Supplement: Additional file 10 — Table S8 Ranking of PIs. Table showing the unshuffled and shuffled relative occurrences for each PI, their difference, and the final ranking based on this difference. [file 1752-0509-4-3-S10.PDF]

Table S8. Ranking of PIs

		unshuffled relative frequency	shuffled relative frequency	difference	rank
ABRWY					
w	A~W	0.106	0.050	0.056	1
w	A~B	0.089	0.037	0.052	2
w	A~Y	0.133	0.101	0.032	3
w	A~R	0.063	0.036	0.027	4
b	A~Y	0.371	0.387	-0.016	5
b	A~R	0.080	0.110	-0.030	6
b	A~B	0.057	0.115	-0.058	7
b	A~W	0.102	0.164	-0.062	8
ABRW+Y					
w	A~W	0.071	0.035	0.036	1
w	A~B	0.059	0.027	0.032	2
b	A~Y	0.543	0.512	0.031	3
w	A~Y	0.089	0.072	0.017	4
w	A~R	0.042	0.026	0.016	5
b	A~R	0.061	0.091	-0.030	6
b	A~B	0.048	0.093	-0.045	7
b	A~W	0.087	0.144	-0.057	8
ABRWY+Y					
w	A~W	0.073	0.038	0.035	1
w	A~B	0.061	0.028	0.033	2
b	A~Y	0.564	0.540	0.024	3
w	A~Y	0.092	0.075	0.017	4
w	A~R	0.044	0.027	0.017	5
b	A~R	0.055	0.084	-0.029	6
b	A~B	0.039	0.086	-0.047	7
b	A~W	0.070	0.123	-0.053	8
ABRWY+Y'					
w	A~W	0.101	0.044	0.057	1
w	A~B	0.084	0.033	0.051	2
w	A~Y	0.127	0.090	0.037	3
w	A~R	0.060	0.032	0.028	4
b	A~R	0.076	0.099	-0.023	5
b	A~B	0.054	0.103	-0.049	6
b	A~Y	0.400	0.450	-0.050	7
b	A~W	0.097	0.148	-0.051	8
