# Supplementary material for: Deciphering chemotaxis pathways using cross species comparisons
Source: BMC Syst Biol. 2010 Jan 11;4:3. doi: 10.1186/1752-0509-4-3 (PMC2829493; doi:10.1186/1752-0509-4-3)
Supplement: Additional file 11 — Figure S1 Chemotaxis pathway of E. coli. Known and predicted core chemotaxis pathways in E. coli [file 1752-0509-4-3-S11.PDF]

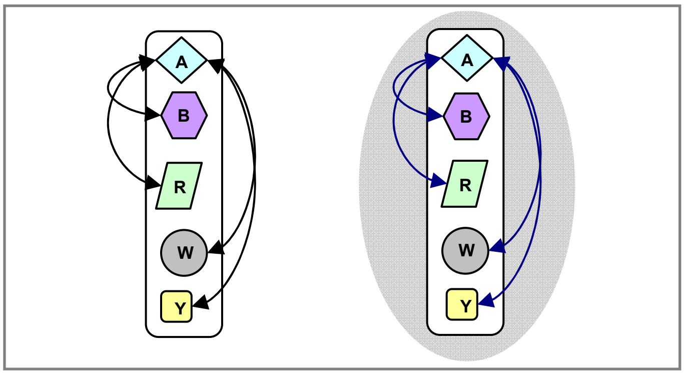

Figure S1. Core chemotaxis pathway of *E.coli.* Left: known pathway, right: pathway predicted by all 4 models. Note this figure does not include CheZ.
