# Supplementary material for: Deciphering chemotaxis pathways using cross species comparisons
Source: BMC Syst Biol. 2010 Jan 11;4:3. doi: 10.1186/1752-0509-4-3 (PMC2829493; doi:10.1186/1752-0509-4-3)
Supplement: Additional file 12 — Figure S2 Chemotaxis pathways of Pseudomonas aeruginosa. Known and predicted chemotaxis pathways in Pseudomonas aeruginosa [file 1752-0509-4-3-S12.PDF]

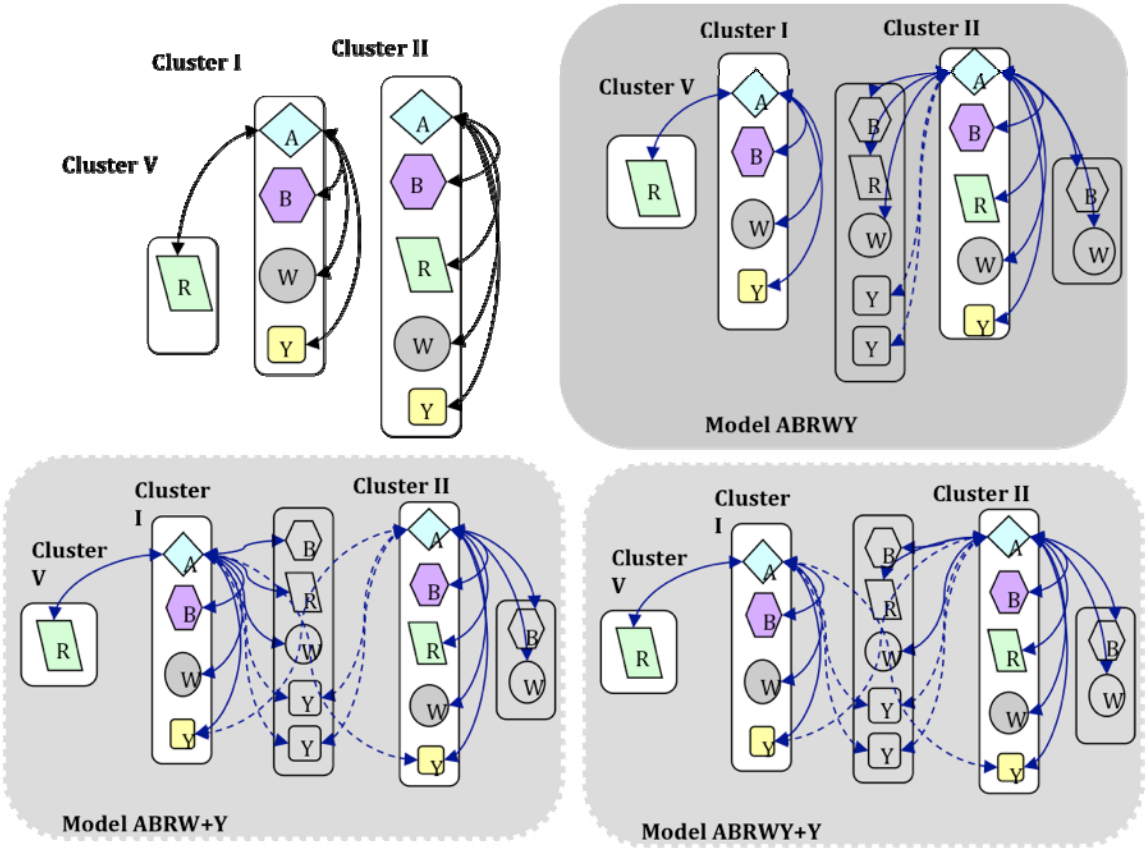

Figure S2. Chemotaxis pathways of *Pseudomonas aeruginosa*
The known pathways (top left) and the predicted pathways (shadowed background) in *P.aeruginosa* from our 4 models are shown. The dotted lines indicate the predicted interactions between CheA and CheY.
