# Supplementary material for: Deciphering chemotaxis pathways using cross species comparisons
Source: BMC Syst Biol. 2010 Jan 11;4:3. doi: 10.1186/1752-0509-4-3 (PMC2829493; doi:10.1186/1752-0509-4-3)
Supplement: Additional file 13 — Table S9 Statistics for R. sphaeroides CheW alignments. Table showing the alignment statistics derived from bl2seq for alignment of each of the 4 CheW homologues in R. sphaeroides and for each of these 4 homologues aligned to E. coli CheW. [file 1752-0509-4-3-S13.PDF]

Table S9. Statistics from blast2seqs for *R. sphaeroides* CheW alignments

Alignment	% identity	% positive	%gaps
CheW1-CheW2	33	51	2
CheW1-CheW3	31	52	3
CheW1-CheW4	33	55	1
CheW2-CheW3	35	52	0
CheW2-CheW4	31	55	0
CheW3-CheW4	28	47	4
CheW1-*E. coli* CheW	34	61	0
CheW2-*E. coli* CheW	25	46	1
CheW3-*E. coli* CheW	33	53	6
CheW4-*E. coli* CheW	41	64	1
