# Supplementary material for: Deciphering chemotaxis pathways using cross species comparisons
Source: BMC Syst Biol. 2010 Jan 11;4:3. doi: 10.1186/1752-0509-4-3 (PMC2829493; doi:10.1186/1752-0509-4-3)
Supplement: Additional file 14 — Figure S3 Alignment of CheA/CheW binding region of MCPs and TLPs. Alignment of the region at the tip of the all 9 MCPs, tlpL and tlpT from R. sphaeroides. [file 1752-0509-4-3-S14.PDF]

```
gi|5420451|MCPG   IHVVQEIARQTDLLALNAAVEAARAGEHGRGFAVVATEVRRLAERSQAAAAEISDLSSTT
gi|77388220|TlpL  INVLREIARQTDLLALNAAVEAARAGQHGAGFSVVAAEVRKLAEHAAAASHEIDQLAHTT
gi|7532754|MCPB   IGIVQEIARQTDLLALNAAVEAARAGEHGRGFAVVAAEVRKLAERSRAAATEISVLSAAT
gi|77389515|MCPH  ILIVQEIARQTDLLALNAAVEAARAGEHGRGFAVVASEVRKLAERSQAAAAEISALSART
gi|77465121|MCP1  ILVVQEIARQTDLLALNAAVEAARAGEHGRGFAVVAAEVRKLAERSRGAAEEISALSART
gi|77465434|MCP2  IGVVREIARQTDLLALNAAVEAARAGEQGRGFAVVAAEVRRLAERSAEAAAEISTLSAGT
gi|8250660|MCPJ   IMMVQEIARQTDLLALNAAVEAARAGEHGRGFAVVASEVRKLAERSQAAAAEISQLSAGT
gi|21436675|TlpT  IKVIDEIAFQTNLLALNAAVEAARAGQHGRGFAVVAHEVRNLAGRSAKAARETSELIEDA
gi|7532756|MCPA   VKVIDDIAFQTNLLALNAGVEAARAGDAGRGFAVVASEVRALAQRSSEAAREITDLILKS
gi|77388204|MCPM  VSFIEGIARKTDLLALNAAVEAARAGEAGRGFAVVAEEVRKLAALTTSSTGEIAAEVGAV
gi|77465422|MCP3  ILVMQEIARQTDLLALNAAVEAARAGERGLGFAVVASEVRKLAERSQDAAKEVSTLSHST
                  : .:  ** :*:******.*******: * **:*** *** **  :  :: *
```

Figure S3. Alignment of the putative CheA/CheW binding region of all 9 MCPs, tlpL and tlpT from *R. sphaeroides*. This region is at the tip of the receptors.
